# Case report: Endoluminal removal of a retrievable conical inferior vena cava filter with a ruptured retraction hook attached to the wall

**DOI:** 10.3389/fsurg.2022.985060

**Published:** 2022-11-10

**Authors:** Xuan Tian, Jianlong Liu, Jinyong Li, Xiao Liu

**Affiliations:** Department of Vascular Surgery, Beijing Jishuitan Hospital, Beijing, China

**Keywords:** inferior vena cava (IVC), filter retrieval, complex, complications, case report

## Abstract

We report the case of a patient who underwent endovascular retrieval of a conical inferior vena cava (IVC) filter with a ruptured retraction hook that was attached to the IVC wall. A 21-year-old woman with a Celect (Cook) filter, implanted 1,522 days prior, requested retrieval. Preoperative ultrasound and CT examinations showed that the filter was inclined, the retraction hook was attached to the IVC wall, and one of the filter’s pedicles was broken. The inferior vena cava was patent, with no thrombus. Old superficial femoral vein thrombosis could be seen in the right lower extremity. The filter retrieval equipment (Gunther Tulip, Cook) failed to capture the retraction hook. By means of a pigtail catheter (with a partly removed catheter tip) and loach guidewire, we applied a modified loop-snare technique to successfully cut the proliferative tissue near the tip of the retraction hook, by which the hook re-entered the inferior vena cava. Although the snare successfully captured the retraction hook and retrieved the filter, the broken pedicle was retained in the inferior vena cava. We used forceps to capture and pull it to the distal end. In the end, the inferior vena cava became patent, with no contrast agent spillage or residual, and no symptomatic pulmonary embolization. A simultaneous occurrence of oblique adherence and fracture is rarely found in the same filter; however, by using the modified loop-snare technique and biopsy forceps technique, we successfully retrieved the filter and broken pedicle. Our case provides a practical auxiliary technique for regular clinical practice.

## Introduction

The American College of Chest Physicians’ (ACCP) 2016 guidelines for the management of venous thromboembolism state that an inferior vena cava (IVC) filter should be removed immediately once the risk of pulmonary embolism has decreased (1–3). Endovascular retrieval of the filter is the first-line option in current clinical practice. When endovascular retrieval fails or complications occur, open surgery is employed to remove the filter. The main reasons for failure are (4, 5) as follows: (1) The filter is severely tilted. Tilt is defined as an angulation of more than 15° from the filter’s long axis; its incidence rate is about 3%–9% (6). When the retraction hook adheres to the IVC wall and is surrounded by proliferative tissue, it cannot be captured, which can result in penetration of the blood vessel wall and damage to the adjacent tissue by the filter retraction hook or pedicle. (2) Fracture of the filter, which occurs at an incidence rate of 1.1%–12.5% (6–8). Filter fractures prevent the filter from being completely removed and increase the risk of filter displacement and broken filter pedicles. Herein, we report the case of a patient who underwent endovascular retrieval of a conical retrievable filter and experienced the two complications mentioned above.

## Case report

Here, we report the case of a 21-year-old woman who requested retrieval of a Celect IVC filter, which had been implanted 1,522 days ago. The patient had been in a traffic accident more than 4 years ago, which had caused pelvic fracture, multiple fractures of the lower extremity, rib fracture with pleural effusion, and hemorrhage in the subarachnoid space. She had been treated with multiple surgeries. Due to deep vein thrombosis in the right lower extremity, the Celect filter was implanted (via left common femoral vein access) to prevent fatal pulmonary embolization. She also underwent open internal fixation surgery for unstable pelvic fractures and closed intramedullary nailing for femoral shaft fractures. The patient had been discharged without postoperative anticoagulation therapy due to factors such as a low hemoglobin count (7.2 g/L), subarachnoid hemorrhage, and pleural effusion. She returned to the local high school to continue her education, and no follow-up had been done in the last 4 years.

The patient complained of dull pain in the right lower abdomen 3 months before admission. Upon admission, venous ultrasound showed an old superficial femoral vein thrombosis in the right lower extremity, but no thrombosis was found in the inferior vena cava. An enhanced abdominal CT also showed that the inferior vena cava was patent, with no hematoma visible. However, the filter was severely inclined, the retraction hook was adhered to the vena cava wall, the main pedicle of the filter was broken, and the broken pedicle was embedded in the wall of the inferior vena cava. Due to the adherence of the retraction hook to the wall and the presence of the broken pedicle, the preferred treatment options were to retrieve the filter by the endovascular method with minimal invasiveness or retrieve the filter laparoscopically (9). Open surgical removal of the filter could also be considered as an alternative if the endovascular therapy failed. After fully balancing the pros and cons, the patient and her family agreed to the less-invasive endovascular therapy.

**Figure 1:**
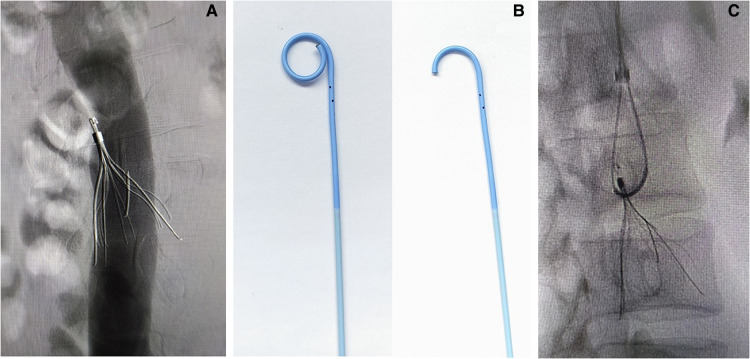
(**A**) The attachment of the Celect filter retrieval hook to the wall of the inferior vena cava, the surrounding proliferative tissue, and a broken pedicle. (**B**) The shape of the pigtail catheter after the removal of the tip. (**C**) Modified loop-snare technique.

**Figure 2:**
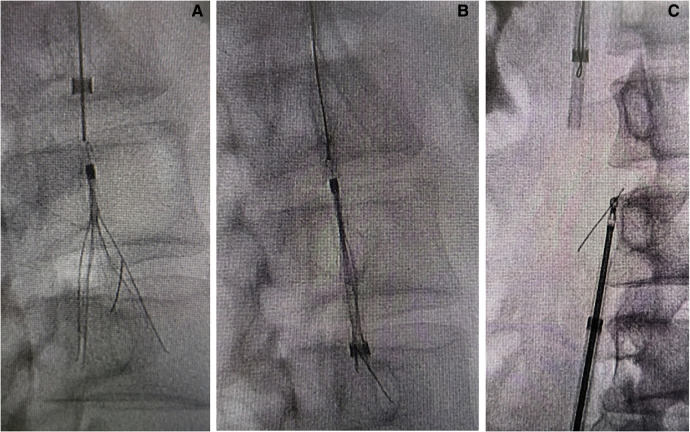
(**A**) Cutting the proliferative tissue surrounding the retraction hook and bringing the hook back into the cavity. (**B**) The filter was captured by the snare, but one broken pedicle remained in the inferior vena cava. (**C**) Successful capture of the fractured pedicle using biopsy forceps.

Angiography via the right common femoral venous access showed that no thrombosis was found in the inferior vena cava, and that it was patent; it also showed that the main pedicle of the Celect filter was broken, the filter was obliquely attached to the retraction hook, and proliferative tissue surrounded the hook (Figure 1A). A filter retrieval set was introduced via the right internal jugular vein; however, it failed to capture the retraction hook. Next, part of the tip (Figure 1B) of the PIG catheter (Cordis; 4F, 100 cm) was cut, and a hydrophilic metal guidewire (Terumo angled, 260 cm) was used to increase the support force of the catheter. Based on a modified loop-snare technique (10) (Figure 1C), the catheter was rotated to be guided into the gap between the filter and the inferior vena cava wall, and the guidewire was advanced to free its end to be snared and externalized. A wire loop was formed across the proliferative tissue, and the counteracting forces of the guidewire and the retrieval sheath were used to destroy the proliferative tissue surrounding the retraction hook (Figure 2A), following which the filter could re-enter the inferior vena cava. Although the snared guidewire captured the retraction hook and retrieved the filter, it failed to capture the broken pedicle, which could not be retrieved simultaneously (Figure 2B). The broken pedicle was therefore retained in the inferior vena cava wall.

**Figure 3:**
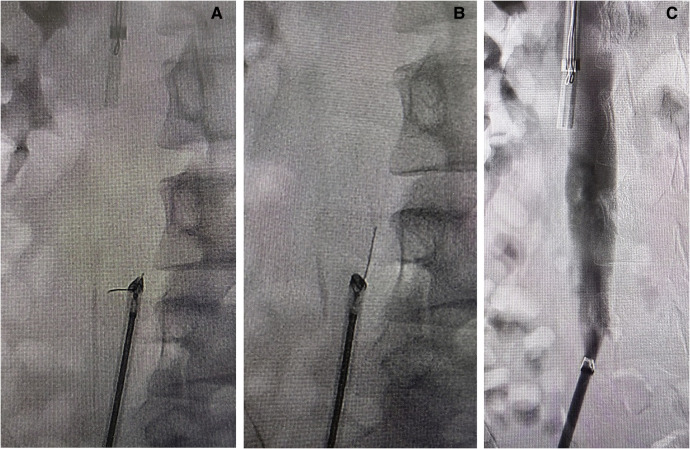
(**A**) The fractured pedicle at a vertical angle to the retrieval sheath and failure of removal. (**B**) The biopsy forceps and the retrieval sheath were pulled toward the distal end to coaxial lines and the broken pedicle was successfully removed. (**C**) The inferior vena cava was unobstructed, without spillage of the contrast agent.

The filter retrieval set and biopsy forceps were introduced via the right common femoral venous access, and the broken pedicle was successfully captured (Figure 2C). However, the pedicle was at a vertical angle to the retrieval sheath and failed to enter it (Figure 3A). The retrieval was unsuccessful after two successive attempts, and the broken pedicle remained at risk of falling off from the inferior vena cava wall.

The broken pedicle was captured again, and the biopsy forceps and the retrieval sheath were used to pull the broken pedicle to the distal end. Then, the angle and coaxial distance were gradually reduced. The broken pedicle was entered into the retrieval sheath (Figure 3B) and was removed. The patient experienced abdominal pain when the pedicle was detached from the inferior vena cava wall. Subsequent angiography showed that the inferior vena cava became patent, with no spillage or residual contrast agent, and the filter was therefore completely retrieved (Figure 3C).

No symptomatic pulmonary embolization occurred during the perioperative period, and the patient received oral rivaroxaban 20 mg QD as an anticoagulation therapy (11) postoperatively. No recurrence of deep vein thrombosis in the lower extremity, no thrombosis in the inferior vena cava, and no symptomatic pulmonary embolism were found during the 3-month follow-up.

## Discussion

The possible complications of long-term IVC filter implantation include obstruction and perforation of the vena cava and approaching or entering of the filter pedicle into the surrounding organs, which can manifest with associated symptoms (12–15). When the IVC filter creates serious complications, it affects patients’ physiological and psychological status (16, 17), and the filter should be retrieved immediately to reduce complications.

Due to abnormalities in the inferior vena cava, the presence of thrombus blocked by the filter, the consequences of the IVC filter placement, or other unknown reasons, the conical filter is more prone to adhere obliquely. Furthermore, the attachment of the retraction hook to proliferative tissues can easily result in failure to capture the hook. The following methods could increase the chances of successfully retrieving the filter: (1) Use of the loop-snare technique (18) to form a wire loop that passes through the filter and is used to correct the filter tilt; (2) Use of a modified loop-snare technique (10), as described in the present case report; (3) Use of double-wire lassoing (19) to form wire loops through the filter at both ends to correct the filter tilt; (4) Use of the biopsy forceps technique (10) to capture the filter, retraction hook, and pedicle, which increases the filter retrieval rate.

The fracture rate of the Celect filter is only 1.1%, which is the lowest rate among all kinds of IVC filters (8). Nevertheless, it was found that the occurrence of filter fracture was related to the filter indwelling time, and that fracture rate increased to 30.8% after more than 3 years of filter indwelling. Tunner et al. (20) reported that 190 filter retrievals with severe complications were conducted in multiple centers, with 90 of the filters removed by endovascular surgery and 100 retrieved by open surgery. Compared with endoluminal filter removal, open surgery had a higher incidence rate venous thromboembolism complications and a 5% mortality rate. Endovascular filter retrieval had a better safety record and a lower rate of complications.

The patient in our study experienced two complications: oblique adherence and fracture of the filter. The modified loop-snare technique and biopsy forceps technique were used to resolve these complications, with an initial failure to capture the retraction hook and retrieve the broken pedicle and filter. There are few similar reports in the recent literature. In contrast to the use of a reverse-curve catheter, as reported by Desai et al. (10), the loop-snare operation in the present case was performed by using a pigtail catheter after partly removing the catheter tip, which had some advantages: (1) The removal site of the pigtail catheter tip could be adjusted according to the position of the retrieval sheath, the direction of inclination, and the shape of the filter, by which the loop-snare technique was applied easily. (2) The medical cost of using the pigtail catheter was relatively low. Both the loop-snare technique and the modified loop-snare technique could be performed using the pigtail catheter, and the guidewire was used only for the purpose of guidance during the operation. As a result, the catheter tip did not damage the vein wall. Furthermore, it was easier to use forceps to capture the retained filter pedicle, compared with the snare method. Initially, the pedicle was at a vertical angle to the retrieval sheath, and the retrieval sheath was unable to acquire the pedicle. We modified the method so that the biopsy forceps and the retrieval sheath were pulled to the distal end simultaneously, and the fractured pedicle was removed successfully after being placed coaxially with the fractured pedicle. (3) The first step was to retract the guidewire into the pigtail catheter; the pigtail catheter could then be easily rotated to better facilitate entry into the gap between the filter and the inferior vena cava wall.

Our surgical method has the following limitations: (1) The head of the pigtail catheter is not robust, and it is therefore easily bent or deformed. The guidewire can be introduced into the pigtail catheter end to increase the supporting force (note, however, that it does not extend to the distal end); (2) The presence of a gap between the filter and the inferior vena cava wall is critical to the success of the modified loop-snare technique. When the filter is severely tilted or deformed, there may be no gap to allow the guidewire and catheter to pass through, making the procedure less successful. (3) A preoperative enhanced CT of the abdomen is a good way to evaluate and initially select the loop-snare technique.

It should be noted that the technique of grasping a broken pedicle using biopsy forceps cannot be performed routinely, and there is a risk of the pedicle falling off. A balloon can be used to block the inferior vena cava at the proximal end, which may reduce the risk of the broken pedicle falling off into the pulmonary artery.

Longer retention times can spell more serious damage for the inferior vena cava when the filter is retrieved, especially if any complications such as a broken pedicle are present. Regular postoperative anticoagulation therapy and follow-up are therefore indispensable. In accordance with the ACCP guidelines (1, 2, 11), we chose regular anticoagulation therapy for 3 months for the patient in the present report. Before discontinuing anticoagulation therapy, an imaging scan of the inferior vena cava and an assessment of the risk of recurrence of thrombosis are recommended. Unfortunately, the patient reported no adverse events during the follow-up call.

In summary, oblique adherence and fracture of conical IVC filters are common complications, but the simultaneous occurrence of both in the same filter is rare. The filter and broken pedicle were removed successfully using a modified loop-snare technique and biopsy forceps technique, avoiding the major trauma of open surgery. Our method can therefore serve as an auxiliary technique for regular clinical practice.

## Data Availability

The original contributions presented in the study are included in the article/Supplementary Material, further inquiries can be directed to the corresponding author/s.
